# NCAPD3‐mediated AKT activation regulates prostate cancer progression

**DOI:** 10.1096/fba.2024-00073

**Published:** 2025-01-07

**Authors:** Yi Zhang, Wanlin Xie, Xicui Zong, Yuanyuan Fang, Jia Ren, Zuolei Jing, Yong Wei, Shan Lu, Qingyi Zhu, Ping Liu

**Affiliations:** ^1^ College of Life Sciences Nanjing Normal University Nanjing Jiangsu China; ^2^ Department of Basic Medicine Nanjing University of Chinese Medicine Hanlin College Taizhou Jiangsu China; ^3^ Department of Urology The Second Affiliated Hospital of Nanjing Medical University Nanjing Jiangsu China

**Keywords:** AKT, NCAPD3, phosphorylation, prostate cancer, STAT3

## Abstract

Despite therapeutic improvements in prostate cancer treatment, the recurrence and mortality rates are still high, and the underlying mechanisms still need further study. Non‐SMC Condensin II Complex Subunit D3 (NCAPD3) is a subunit of condensin II complex, mainly involved in the mitotic chromosome condensation of cells. This study aimed to figure out the detailed mechanisms by which NCAPD3 contributed to prostate cancer development. Clinical samples and cell lines were used to measure the expression of genes by quantitative real‐time RT‐PCR (qRT‐PCR), Western‐blot assay (WB), immunohistochemistry (IHC), and immunofluorescence (IF). Chromatin immunoprecipitation quantitative PCR (ChIP‐qPCR) and dual‐luciferase reporter assays were examined to explore the interplays between molecules. CCK8, transwell, and wound‐healing assays were applied to perform cell proliferation and migration. A subcutaneous tumor xenograft model was constructed by injecting DU145‐Lv‐NCAPD3 cells and control cells into male BALB/c nude mice to confirm the result derived from in vitro assay. NCAPD3 increased STAT3 expression and phosphorylation in PCa cells, thereby enhancing STAT3 transcriptional activity to improve the levels of JAK2 and EZH2. This led to an increase in phosphorylation of AKT at Thr 308 and Ser 473 through JAK2/PI3K and EZH2/NSD2/mTORC2 pathways, respectively. Moreover, there was a positive mutual activation between STAT3 and JAK2, further enhanced by NCAPD3 to promote PCa progression. NCAPD3, as an oncogene, promoted PCa progression by phosphorylating and activating AKT, which suggests a novel functional pathway of NCAPD3 in promoting PCa progression.

## Graphical abstract

Abnormally expressed NCAPD3 in prostate cancer caused STAT3 to be highly expressed and bound to the promoters of JAK2 and EZH2 to promote their transcription, increasing the level of AKT phosphorylation, and accelerating tumor development. In addition, since JAK2 has been shown to activate STAT3, there was reciprocal activation of JAK2 and STAT3 in this process.

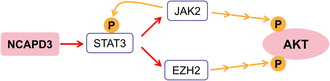

## 1 INTRODUCTION

Prostate cancer (PCa) is the most commonly diagnosed malignancy and the second leading cause of cancer death in Western countries.^1^, ^2^ In addition to ethnic differences and age factors, many genetic and epigenetic changes are considered to be tumor drivers associated with PCa, including phosphatase and tensin homolog (PTEN) deletion, SPOP mutation, TMPRSS2‐ERG translocation, and Myc amplification.^3^ The complexity of molecular mechanisms in the occurrence and progression of PCa always induces castration resistance and disease recurrence after androgen deprivation therapy (ADT) in the clinic.^4^ Thus, more studies on the molecular mechanism are urgently needed to provide evidence for finding better clinical diagnosis and treatment of PCa.

Condensin complexes, including condensin I and condensin II, play fundamental roles in chromosome organization and segregation during cell mitosis. In humans, each condensin complex consists of five subunits: two structural maintenance of chromosomes (SMC) subunits (SMC2 and SMC4) and three unique non‐SMC subunits (condensin I contain NCAPD2, NCAPG, and NCAPH; condensin II contain NCAPD3, NCAPG2, and NCAPH2).^5^, ^6^ Mutation of NCAPD3 causes abnormal mitotic cells and microcephaly, suggesting that mitotic chromosome condensation is a key process to ensure the size of the mammalian cerebral cortex.^7^ In addition, some studies have shown that NCAPD3 is related to various intracellular physiological processes, including gene expression regulation, DNA damage repair, histone modification, cell respiration, and oxidative stress.^8^, ^9^ In pancreatic ductal adenocarcinoma, NCAPD3 is a new predictive prognostic factor.^10^ Recently, the important functions of NCAPD3 in diseases, especially in cancers, have gradually attracted the attention of researchers.

AKT (also known as protein kinase B) is a serine/threonine kinase that includes three isoforms (AKT1, AKT2, and AKT3) in the mammalian genome.^11^ Studies have shown that AKT is critical in regulating cell survival, insulin signaling, angiogenesis and tumorigenesis, and its overexpression makes cancer cells resistant to cisplatin.^12^ In molecular mechanism, AKT phosphorylates pro‐apoptotic proteins such as Bad, FOXO1, and FOXO3A to prevent cell apoptosis, while phosphorylates many oncoproteins such as MDM2, IKKα, and Skp2 to promote cell cycle progression and tumorigenesis.^13^, ^14^ Although JAK2/PI3K and EZH2/NSD2/mTORC2 axes are all the upstream signal pathways of AKT phosphorylation in many types of cancers,^15^ the relationships among them in PCa are not clear.

In this study, in vitro and in vivo experimental data confirmed that NCAPD3 was highly expressed in prostate cancer, which could activate AKT and promote PCa progression. In detail, NCAPD3 increased JAK2 and EZH2 by inducing STAT3‐mediated transcriptional activation to enhance the phosphorylation of AKT at Thr308 and Ser 473. We also found that NCAPD3 enhanced the positive mutual activation between STAT3 and JAK2. Therefore, our study highlighted a novel important mechanism of NCAPD3 in promoting the progression of PCa.

## 2 MATERIALS AND METHODS

### 2.1 Cell culture and transfection

Cell lines used in this study included human normal prostate epithelial cell line WPMY‐1, human prostate hyperplasia cell line BPH‐1 and PCa cell lines (PC‐3, DU145, 22Rv1, LNCaP), which were purchased from the American Type Culture Collection Center (Manassas, VA, USA). WPMY‐1 were cultured in Dulbecco's Modified Eagle's Medium (DMEM; Gibco, Grand Island, NY, USA) containing 10% fetal bovine serum (FBS; Gibco), The other cell lines were cultured in Roswell Park Memorial Institute 1640 (RPMI‐1640; Gibco) containing 10% fetal bovine serum. All cells were maintained in a humidified incubator at 37°C with 5% CO_2_. For transfection experiments, cells were seeded in a 6‐well plate until 70%–80% densities, and the corresponding siRNAs or plasmids were transfected with Lipofectamine 2000 (Invitrogen, CA, USA). siRNAs used in this study were purchased from GenePharma (Shanghai, China) and the relevant information is listed in Table S1.

### 2.2 Antibodies, reagents, and Western‐blot assay

Primary antibodies against NCAPD3 (Cat#16828), FoxO1 (Cat#18592), FoxO3A (Cat#10849), STAT3 (Cat#10253), EZH2 (Cat#21800), GAPDH (Cat#10494), PI3K p110β (Cat#20584), PTEN (Cat#60300), JAK2 (Cat#17670), mTOR (Cat#66888), P70S6K (Cat#14485), p‐P70S6K(T389) (Cat#28735), and H3K27me3 (Cat#61017) were from Proteintech (Wuhan, China). Antibodies against NSD2 (Cat#sc‐365627) and NCAPH2 (Cat#sc‐393333) were from Santa Cruz (California, USA). Antibodies against β‐actin (Cat#AC038), Lamin B (Cat#A11495), mLST8 (A1059), p‐FoxO3A(S253) (Cat#AP0684), 4E‐BP1 (Cat#A24691), p‐4E‐BP1(T70) (Cat#AP1334), and p‐AKT(T308) (Cat#AP1259) were from ABclonal Technology (Wuhan, China). Antibodies against AKT (Cat#9272) and p‐AKT(S473) (Cat#4051) were from Cell Signaling Technology (Massachusetts, VA, USA). Antibodies against p‐FoxO1(Ser256) (Cat#AF3417), RICTOR (Cat#DF7530), RAPTOR (Cat#DF7527), PI3K p85α (Cat#AF6241), p‐PI3K p85α (Tyr607) (Cat#AF3241), and p‐STAT3(Y705) (Cat#AF3293) were from Affinity Biosciences (Cincinnati, OH, USA). The secondary antibodies HRP Goat Anti‐Rabbit IgG (H + L) (Cat#AS014) and HRP Goat Anti‐Mouse IgG (H + L) (Cat#AS003) were from ABclonal Technology. The inhibitors for PI3K (LY294002; Cat#HY‐10108), JAK2 (Z3; Cat#HY‐15480), mTORC2 (Torin1; Cat#HY‐13003), STAT3 (Stattic; Cat#HY‐13818), and AKT (MK‐2206; Cat#HY‐10385) were from MedChemExpress (Newark, NJ, USA) and inhibitor for EZH2 (GSK126; Cat#500561) were from Merck (Shanghai, China).

For Western‐blot assay, cell pellets were suspended in cell lysate (20 mM Tris–HCl pH 8.0, 150 mM NaCl, 2 mM EDTA, 1% NP‐40, 1 mM DTT, 1× protease inhibitor cocktails) for 30 min at 4°C and then centrifugated at 4°C, 14,000 rpm for 15 min. The protein samples were fractionated by SDS‐PAGE and then transferred to a polyvinylidene fluoride membrane. The membrane was subsequently blocked by 5% defatted milk for 1.5 h, incubated with the primary antibody overnight at 4°C, and then followed by incubation with the secondary antibody at room temperature for 2 h. The final result was photographed by using an enhanced chemiluminescence plus detection system.

### 2.3 Quantitative real‐time RT‐PCR (qRT‐PCR)

The total RNA was extracted from cells by using TRIzol reagent (Invitrogen) according to the manufacturer's instructions. The first strand cDNA was synthesized using HiScript® II 1st Strand cDNA Synthesis Kit (Vazyme biotech, Nanjing, China). The cDNA was detected with AceQ qPCR SYBR Green Master Mix (Vazyme biotech). Relative RNA expression was analyzed with the 2‐∆∆ct method (β‐actin level used for standardization). Primer sequences are listed in Table S2.

### 2.4 Cell proliferation and migration assays

Cell viability was measured by Cell Counting Kit‐8 (CCK‐8; Beyotime, Shanghai, China) and EdU kit (BeyoClick™ EdU Cell Proliferation Kit with Alexa Fluor 488, Beyotime) according to the manufacturer's instruction. For colony formation assay, cells (3 × 10^3^ cells/well) with transfections were incubated in 6‐well plates for about 2 weeks, then stained with 0.1% crystal violet solution. The colonies were photographed and counted with microscopy. For wound‐healing assay, the sterile pipette tips were used to scratch a straight line when cells were cultured to 95% confluent, and then washed with PBS. Photographs of the wound area were respectively taken at 0 h and 48 h by microscopy.

For transwell assay, the treated cells (1 × 10^5^ cells/mL) were collected and re‐cultured in the transwell upper chamber (Corning, NY, USA), while 10% FBS medium was added into the lower chamber. After 48 h, cells that had not migrated through the pores were manually removed from the upper face of the filters using cotton swabs, and cells adherent to the lower surface of the filters were fixed in cold 4% (w/v) paraformaldehyde for 30 min and then stained with 0.1% (w/v) crystal violet for 30 min. Finally, the filters were washed thoroughly in 1 × PBS, and the cells were photographed and counted under a microscope with a digital imaging system (Olympus DP50, Olympus, Japan) in the appropriate magnification.

### 2.5 Nuclear and cytoplasmic protein extraction

The nuclear and cytoplasmic proteins were separately extracted by using a nucleocytoplasmic separation kit (Beyotime) according to the manufacturer's instructions. Briefly, cell pellets were lysed with lysis buffer (10 mM Tris–HCl pH 7.4, 0.2 mM MgCl_2_, 1% Triton‐X 100) on ice for 10–15 min. The lysate was then centrifuged for the separation of the nucleoplasm (precipitation) and the cytoplasmic fractions (supernatant). The precipitation was used for further lysis to extract nuclear proteins. All protein samples were finally analyzed by Western blot assay. Lamin B and GAPDH were used as internal controls for the detection of nuclear protein and cytoplasmic protein, respectively.

### 2.6 Dual‐luciferase reporter assay and chromatin immunoprecipitation quantitative PCR (ChIP‐qPCR)

JAK2 promoter was cloned into a pGL‐basic vector to construct the dual‐luciferase reporter plasmid, termed pGL3‐JAK2. DU145 and LNCaP cells were seeded in 6‐well plates and co‐transfected with pGL3‐JAK2, Renilla vector and pcDNA3.1‐NCAPD3 or siSTAT3. After 48 h, the total proteins were extracted by Dual‐Luciferase reporter assay kit (Beyotime), and the dual‐luciferase activities were determined according to the manufacturer's protocol. The relative luciferase activities were calculated and shown in the figures.

The experimental procedures were mainly based on the manufacturer's instructions of ChIP assay kit. Briefly, cells were cross‐linked with formaldehyde, lysed with SDS Lysis buffer and sonicated to shear the chromatin; and then the lysates were centrifuged. The supernatants were collected and incubated with 2 μg of antibody against STAT3 or normal rabbit IgG (as a control) at 4°C overnight. Eluted and purified DNA fragments were then analyzed by real‐time qPCR assays. The supernatants of sonicated lysates were also included as input controls. qPCR was performed using primers specific for the STAT3 binding regions in promoters of EZH2 and JAK2. The details of primers are listed in Table S3.

### 2.7 Immunofluorescence (IF) and immunohistochemistry (IHC)

For immunofluorescence assay, transfected cells were seeded into 6‐well plates with coverslips for 24 h; and then the cells on coverslips were fixed with 4% paraformaldehyde and permeabilized by 0.3% Triton X‐100 for 20 min. After blocking with 1% bovine serum albumin, the cells were incubated with the specific primary antibodies overnight at 4°C and followed by the secondary antibodies and DAPI. Images were captured by Nikon confocal microscope (Nikon; Tokyo, Japan) and analyzed by Image J software.

For IHC assay, the tissues of patients or mice fixed with paraformaldehyde were prepared into paraffin‐embedded sections in advance. The sections were sequentially deparaffinized, hydrated and antigen retrieval, and then incubated with specific primary antibodies overnight at 4°C, followed by detection with the secondary antibody binding by a streptavidin‐biotin peroxidase (Boster Biological Technology, Wuhan, China). The nuclei were counterstained with hematoxylin, then dehydrated and mounted with neutral resin. Images were captured with a microscope and the positive density was measured by Image J software.

### 2.8 In vivo subcutaneous tumor assay

Male 6‐week‐old BALB/c nude mice were purchased from the Model Animal Research Center of Nanjing University (Nanjing, China). All animal studies were approved by the Nanjing Normal University Animal Ethics Committee. All mice were housed under special pathogen‐free conditions, and the room was maintained at a controlled temperature (21 ± 1°C) and humidity (55 ± 10%) with illumination at 12‐h cycles; food and water were available ad libitum. The NCAPD3‐stable transfection DU145 (2 × 10^6^ cells) were suspended with 100 μL PBS and subcutaneously injected into the left flank of mice (5 mice per group). The body weight and tumor size were measured every 2 days. The formula V = 0.5ab^2^ (a, the tumor length; b, the tumor width) was applied to calculate tumor volumes. Mice were humanely euthanized by cervical dislocation under ether anesthesia when the volume of the tumor reached 300 mm^3^ (38 days post tumor cell injection), and tumor tissues were dissected and preserved for subsequent assays.

### 2.9 Patient samples

In the present study, adjacent normal tissues and PCa tissues were collected through surgery from the Affiliated Hospital of Nanjing University of Chinese Medicine (Nanjing, China). All patients provided informed consent for collected samples and subsequent analysis. The samples were snap‐frozen in liquid nitrogen or formalin‐fixed and paraffin‐embedded for subsequent experiments. All the experiments were approved by the Ethics Committees of the Affiliated Hospital of Nanjing University of Chinese Medicine. The relevant omics data from Genotype‐Tissue Expression (GTEx), the Cancer Genome Atlas Program (TCGA) database, and Cancer Cell Line Encyclopedia (CCLE) were analyzed.

### 2.10 Statistical analysis

The statistical analyses were performed with SPSS 17.0 and Graphpad Prism 8.0. All experimental data were obtained from three independent experiments at least and showed as Mean ± SD or SEM. Student's *t*‐test was used for comparison within the group, *p < 0.05; **p < 0.01; ***p < 0.001; ns, not significant.

## 3 RESULTS

### 3.1 NCAPD3 potentially promoted prostate cancer development by upregulating STAT3 expression and phosphorylation

**FIGURE 1:**
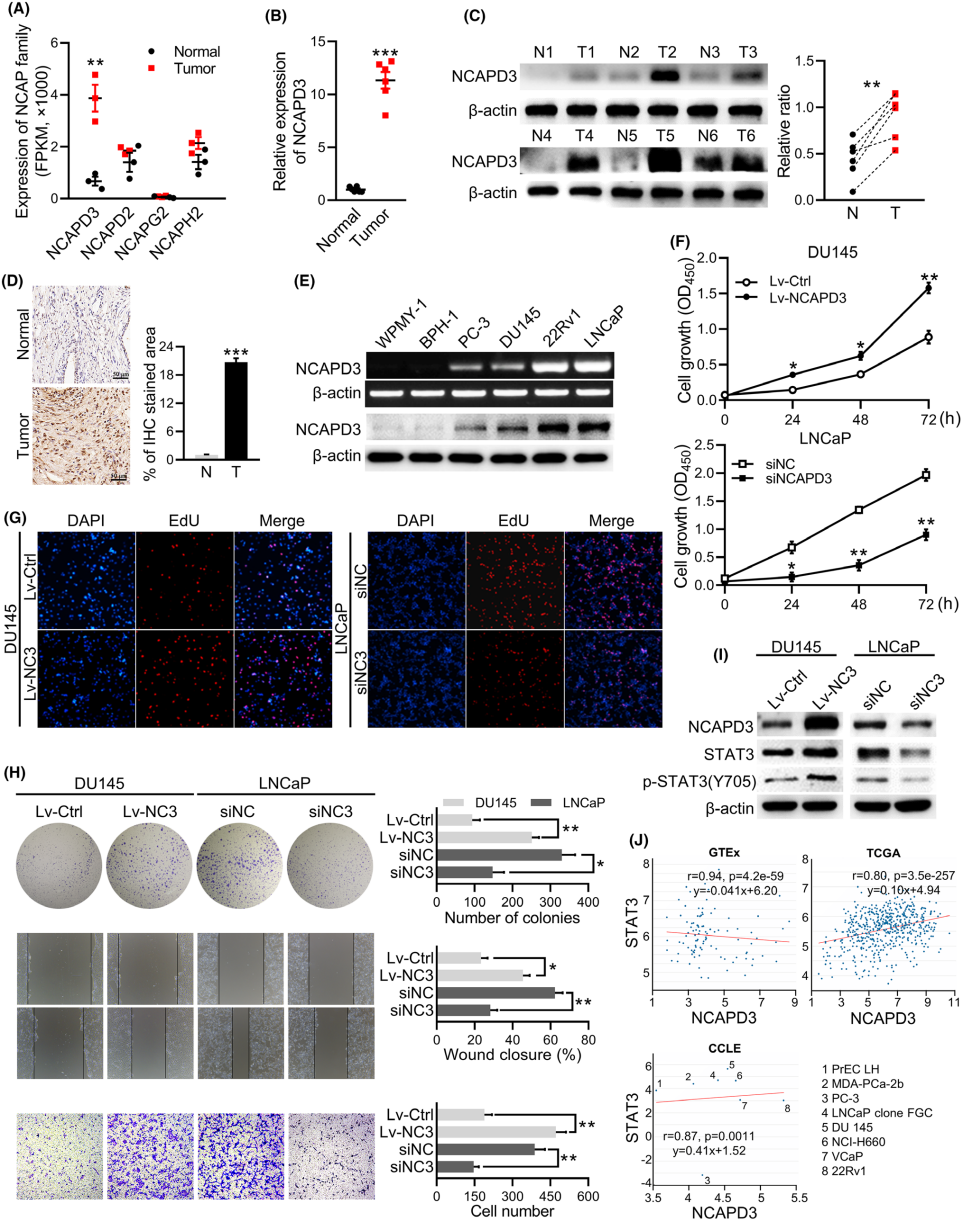
NCAPD3 promoted the development of prostate cancer possibly by upregulating the expression and phosphorylation of STAT3. (A) Relative expressions of the non‐SMC subunits by analyzing our RNA‐seq data of clinical specimens. (B, C) qRT‐PCR (B) and Western blotting analysis (C) for NCAPD3 expression in prostate tumor tissues and adjacent normal tissues. T, tumor tissues; N, adjacent normal tissues. (D) Immunohistochemistry staining of NCAPD3 in clinically normal and PCa specimens. Scale bar: 50 μm. (E) mRNA and protein levels of NCAPD3 in human normal prostate epithelial cell line WPMY‐1, prostate hyperplasia cell line BPH‐1, and PCa cell lines (PC‐3, DU145, 22Rv1, LNCaP) examined by PCR and western blotting. (F–H) Cell growth was assessed by CCK8 assay (F), EdU staining (G), and colony formation (H, upper) in DU145 and LNCaP cells with NCAPD3 overexpression and knockdown, respectively. Cell motility and migration were detected by wound‐healing assay (H, middle) and transwell (H, lower) in DU145 and LNCaP cells with NCAPD3 overexpression and knockdown, respectively. Values are means ± SE from *n* = 3 independent repetitions, *p < 0.05, **p < 0.01, ***p < 0.001, based on Student's *t*‐test. (I) Levels of STAT3 and p‐STAT3(Y705) were checked by Western blot assays in DU145 and LNCaP cells with transfection of NCAPD3 and siNCAPD3, respectively. (J) Correlations analysis between NCAPD3 and STAT3 expression in Genotype‐Tissue Expression (GTEx), the Cancer Genome Atlas Program database (TCGA), and Cancer Cell Line Encyclopedia (CCLE).

RNA‐seq data of clinical specimens showed that NCAPD3 was significantly higher in PCa tissues than adjacent normal tissues, but other detectable non‐SMC subunits, including NCAPD2, NCAPG2, and NCAPH2, did not change significantly (Figure 1A). The levels of NCAPD3 mRNA and protein were significantly higher in PCa tissues compared to normal tissues, as detected by qRT‐PCR, Western blot, and immunochemistry analysis (Figure 1B–D). In human prostate cell lines, NCAPD3 was also higher in all tumor cell lines (PC‐3, DU145, 22Rv1, and LNCaP) than in non‐cancer cell lines (WPMY‐1 and BPH‐1) (Figure 1E). To detect the effect of NCAPD3 on PCa progression, we established stable NCAPD3‐overexpression DU145 cells and transient transfected LNCaP cells with NCAPD3 siRNA, and then evaluated the ability of proliferation and migration in these cells by CCK8 assay, EdU staining, clone formation assay, wound‐healing assay, and transwell assay (Figure 1F–H). It is apparent that NCAPD3 significantly promoted prostate cancer cell viability and migratory capacity.

Much literature has reported that STAT3 has been implicated in promoting the oncogenesis and progression of prostate cancer, and we found that NCAPD3 not only promoted STAT3 expression but also regulated STAT3 phosphorylation (Figure 1I). We found a negative correlation between NCAPD3 and STAT3 in normal prostate samples from Genotype‐Tissue Expression (GTEx), but the strong positive relevance between them in PCa patients from the Cancer Genome Atlas Program (TCGA) database (Figure 1J). In 8 prostate cell lines from Cancer Cell Line Encyclopedia (CCLE), we found that most of the correlations were positive by calculating Pearson's correlation, except PC3 cell line (no STAT3 expression). These results demonstrated that NCAPD3 could promote prostate cancer cell proliferation and migration by STAT3.

### 3.2 NCAPD3 upregulated the activity of AKT by increasing the phosphorylation of AKT in PCa cells

**FIGURE 2:**
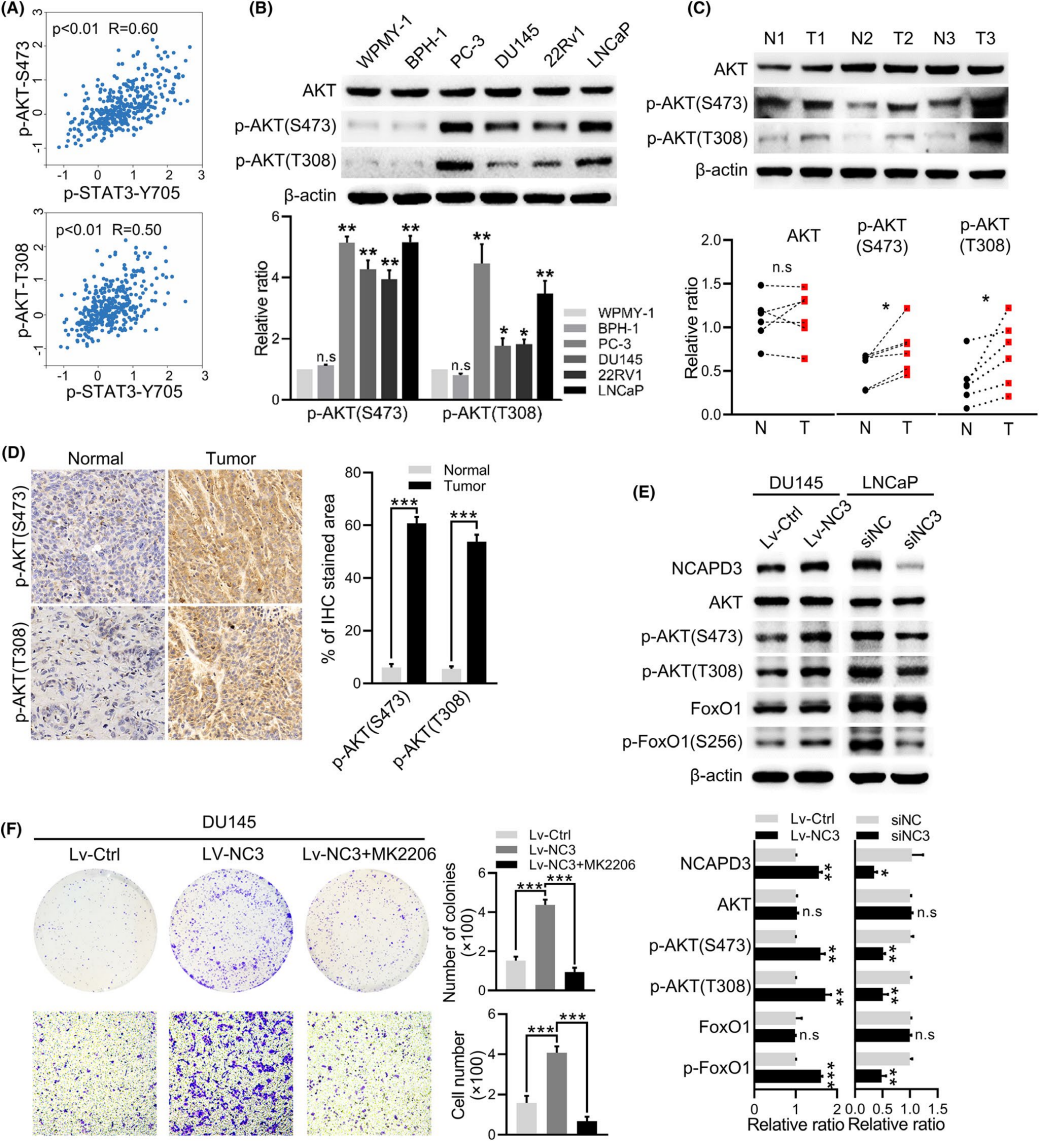
NCAPD3 enhanced the phosphorylation and activation of AKT in PCa. (A) The protein expression of p‐AKT (S473), p‐AKT (T308), and p‐STAT3 (Y705) were retrieved from TCGA Prostate adenocarcinoma database (https://www.tcpaportal.org/). (B) Levels of total AKT, p‐AKT(S473), and p‐AKT(T308) measured by Western blot assay in non‐cancer prostate cell lines (WPMY‐1, BPH‐1) and PCa cell lines (PC‐3, DU145, 22Rv1, LNCaP). (C) Protein expression of total AKT, p‐AKT(S473), and p‐AKT(T308) checked by Western blot assay in clinical tissues. T, tumor tissues; N, adjacent normal tissues. (D) IHC staining of p‐AKT(S473) and p‐AKT(T308) in tumor tissues and matched normal tissues. Scale bar: 50 μm. (E) Western blot assays for the phosphorylation and total protein levels of AKT and FoxO1 in NCAPD3 overexpression and knockdown cells. (F) Colony formation and transwell assays for assessing the cell viability and migration in DU145 cells with NCAPD3 transfection and MK‐2206 (2 μM) treatment, respectively. Values are means ± SE from *n* = 3 independent repetitions, *p < 0.05, **p < 0.01, ***p < 0.001, based on Student's *t*‐test.

Through the analysis of 351 samples from TCGA Prostate adenocarcinoma database using the Cancer Proteome Atlas (https://www.tcpaportal.org/tcpa/), we found that both p‐AKT (S473) and p‐AKT (T308) were positively correlated with p‐STAT3 (Y705) in PCa (Figure 2A). As we known, overactivation of AKT is the most commonly altered pathway in primary PCa, which regulates cell proliferation, migration, and cancer metastasis.^16^, ^17^ This was confirmed in our analysis, phosphorylations of AKT at Thr308 and Ser473 were significantly higher in PCa cells than in non‐cancer cells, while the total protein levels of AKT did not change (Figure 2B). WB and IHC also validated p‐AKT (S473) and p‐AKT (T308) were higher in tumor tissues than normal tissues, but total AKT did not change (Figure 2C,D). Overexpression of NCAPD3 in DU145 cells increased phosphorylation of AKT and its target genes, including FoxO1 and FoxO3A (Figure 2E, Figure S1A). Meanwhile, reverse results were obtained in LNCaP with NCAPD3 knockdown. MK‐2206 (an inhibitor of AKT) significantly impaired NCAPD3‐promoted proliferation and migration of PCa cells (Figure 2F). These results indicated that NCAPD3 may promote prostate cancer by enhancing AKT activity. However, our results in Figure S1B identified that NCAPD3 was mainly located in the nucleus, but AKT and p‐AKT were primarily in the cytoplasm. Thus, the regulation mechanism of phosphorylation of AKT by NCAPD3 should be further investigated.

### 3.3 NCAPD3 enhances the phosphorylation of AKT (T308) via JAK2/PI3K axis

**FIGURE 3:**
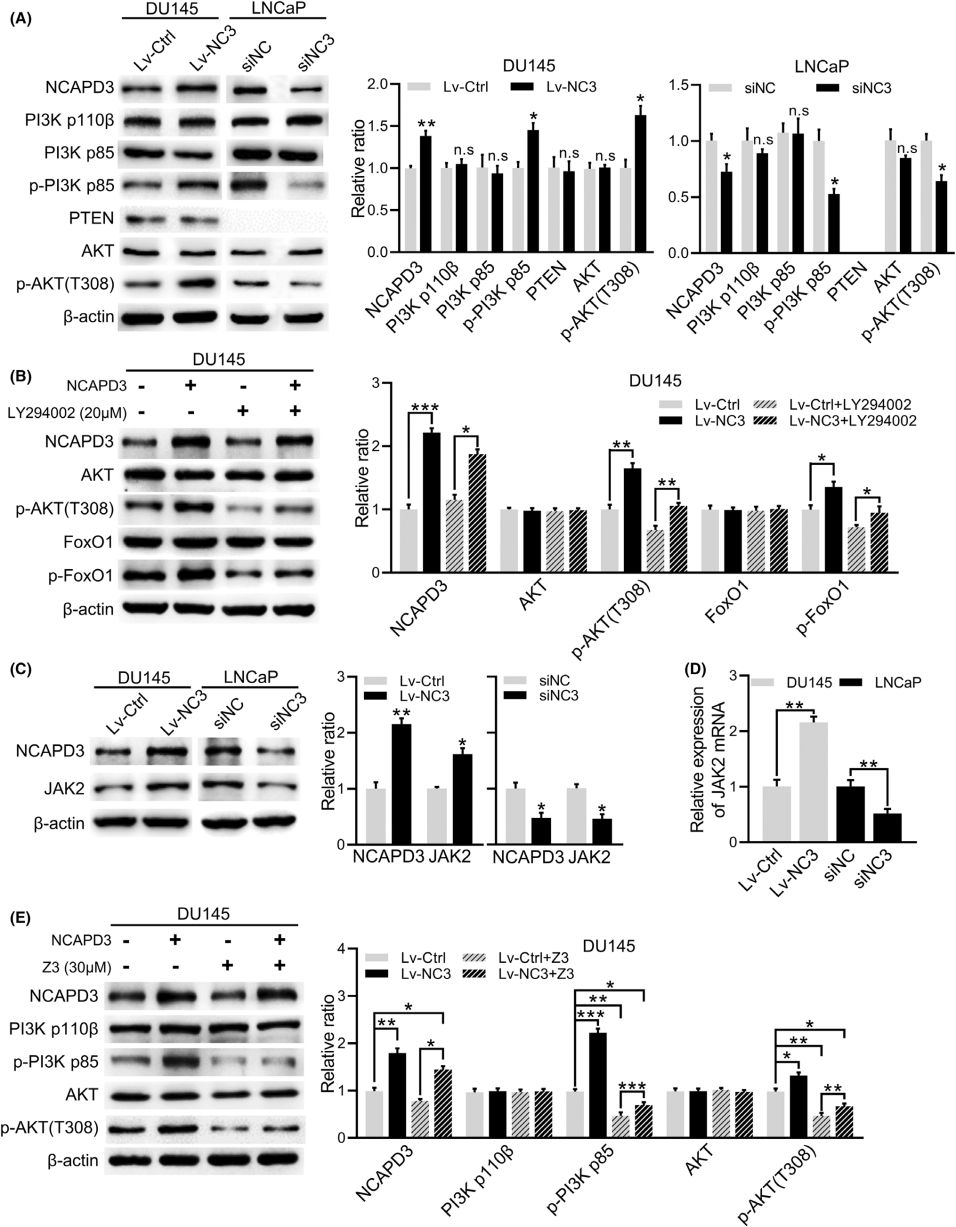
NCAPD3 upregulated the phosphorylation level of AKT (T308) via JAK2/PI3K axis. (A) Protein levels of PI3K p110β, PI3K p85, AKT, PTEN, p‐PI3K p85, and p‐AKT (T308) assayed by Western blot in NCAPD3 overexpression or knockdown PCa cells. (B) Protein expressions of NCAPD3, AKT, p‐AKT (T308), FOXO1, and p‐FOXO1 (S256) in DU145 cells with transfection of NCAPD3 and treatment of LY294002 (20 μM). (C and D) Levels of JAK2 protein and mRNA expressions in NCAPD3‐overexpression DU145 cells and NCAPD3‐knockdown LNCaP cells. (E) Protein levels of NCAPD3, PI3K, p‐PI3K, AKT, and p‐AKT (T308) in DU145 cells with transfection of NCAPD3 and treatment of Z3 (30 μM). Values are means ± SE from *n* = 3 independent repetitions, *p < 0.05, **p < 0.01, ***p < 0.001, based on Student's *t*‐test.

The phosphatidylinositol 3‐kinase (PI3K) is the upstream activator of AKT, which converts PI(3,4)P2 into PIP3 and then promotes AKT phosphorylation at T308.^17^ To prove that PI3K kinase activity was required for AKT T308 phosphorylation enhanced by overexpression of NCAPD3, the level of expression and phosphorylation of PI3K subunits and target genes were detected in PCa cells. As shown in Figure 3A, the phosphorylation levels of PI3K and AKT (T308) were increased in DU145 cells with NCAPD3 overexpression and decreased in LNCaP cells with NCAPD3 knockdown, while the levels of total PI3K and total AKT were not changed. Due to PTEN dephosphorylation activity being critically involved in the regulation of AKT phosphorylation level, we also verified the effect of NCAPD3 on PTEN. NCAPD3 did not change the level of PTEN in both DU145 and LNCaP, so we considered that NCAPD3's effect on p‐AKT (T308) was not mediated via PTEN, and more likely through upstream phosphokinases, like PI3K (Figure 3A). Meanwhile, this might explain why phosphorylations of AKT were lower in PTEN‐expressing DU145 than in PTEN‐null PC‐3 (Figure 2B). Treating with PI3K inhibitor LY294002 in DU145 cells was also demonstrated that NCAPD3‐enhanced phosphorylation of AKT (T308) and FoxO1 (S256) was suppressed when PI3K was blocked (Figure 3B).

As we know, JAK2 activates PI3K/AKT signaling pathway by phosphorylating PI3K.^18^ Our results showed that the expression of JAK2 was increased in DU145 cells with NCAPD3 overexpression and reduced in LNCaP cells with NCAPD3 knockdown (Figure 3C,D). JAK2 inhibitor Z3 blocked phosphorylation of PI3K and AKT (T308) induced by NCAPD3 in NCAPD3‐overexpressed DU145 cells (Figure 3E). These results suggested that NCAPD3 increased the phosphorylation of AKT (T308) via JAK2/PI3K axis.

### 3.4 NCAPD3 enhances the phosphorylation of AKT (S473) via STAT3/EZH2/NSD2 axis

**FIGURE 4:**
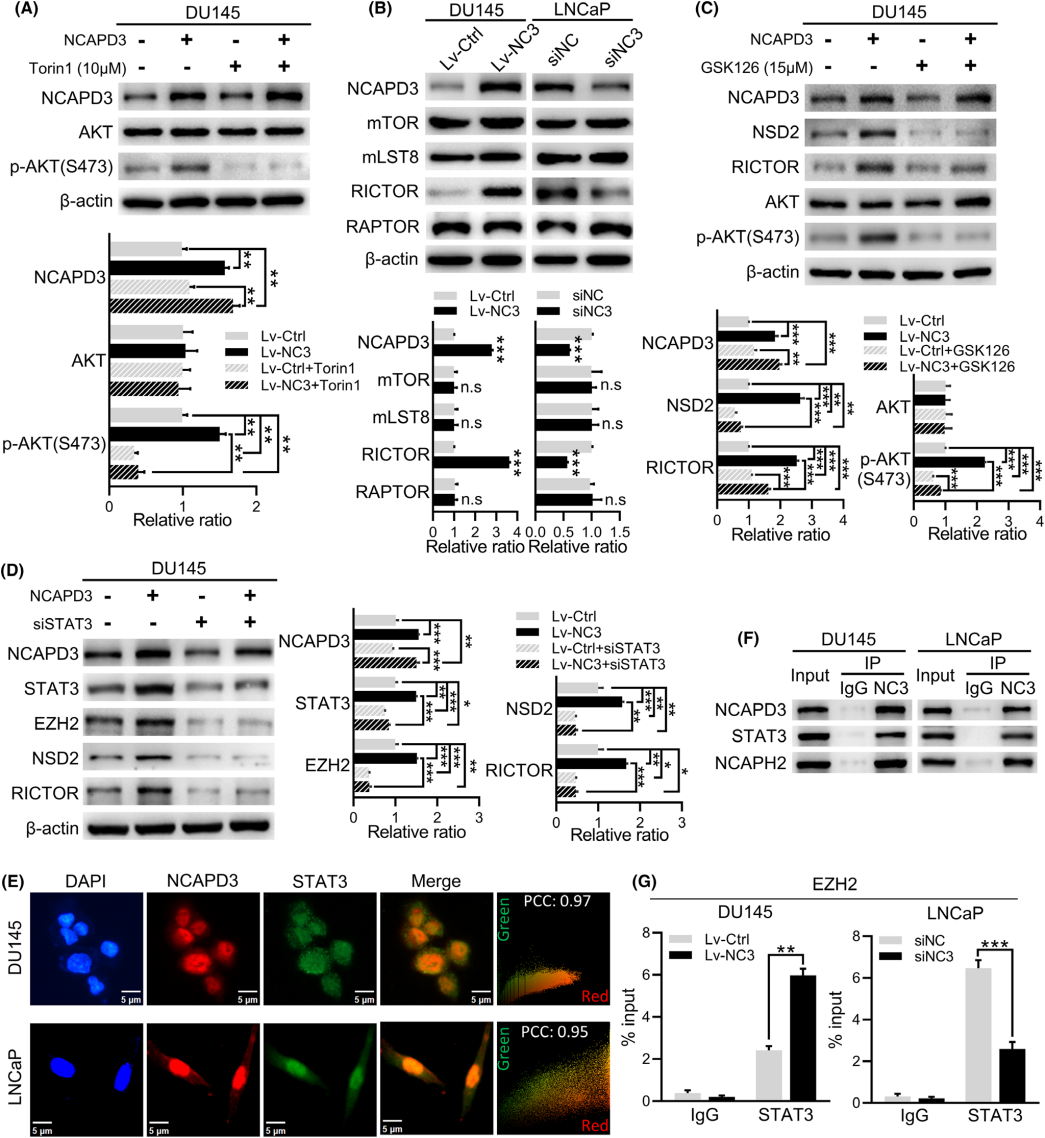
NCAPD3 increased the phosphorylation level of AKT (S473) via STAT3/EZH2/NSD2 axis. (A) Levels of AKT and p‐AKT(S473) in PCa cells with overexpression of NCAPD3 and treatment of Torin 1 (10 μM). (B) Expression of mTOR, mLST8, RICTOR, and RAPTOR in NCAPD3‐overexpressed and NCAPD3‐knockdown PCa cells. (C) Protein levels NCAPD3, NSD2, RICTOR, AKT, and p‐AKT (S473) in NCAPD3‐overexpressed DU145 cells treated with GSK126 (15 μM). (D) Protein levels of NCAPD3, STAT3, EZH2, NSD2, and RICTOR in DU145 cells transfected with NCAPD3 and siSTAT3. (E) Immunofluorescence staining of NCAPD3 (Red), STAT3 (Green) and cell nucleus (Blue) in DU145 and LNCaP cells. Merged images represented overlays of Red and Green. Quantitative analysis of positively stained areas in the same cell nuclei by calculating Pearson's correlation coefficient (PCC) using Image‐Pro Plus software. PCC values close to 1, reflecting high degree of colocalization. (F) Co‐immunoprecipitation (Co‐IP) assays for detecting the interaction between NCAPD3 and STAT3 in PCa cells. (G) ChIP‐assay for evaluating the enrichment of STAT3 in EZH2 promoter in NCAPD3‐overexpression DU145 cells and NCAPD3‐knockdown LNCaP cells, respectively. IgG served as a nonspecific control. Values are means ± SE from *n* = 3 independent repetitions, *p < 0.05, **p < 0.01, ***p < 0.001, based on Student's *t*‐test.

mTORC2 is a critical activator of AKT by promoting the phosphorylation at Ser473.^17^ To identify whether mTORC2 was involved in NCAPD3‐induced hyperphosphorylation of AKT at S473, DU145 cells were treated with mTOR inhibitor (Torin 1) after overexpressing with NCAPD3. Torin 1 significantly suppressed the NCAPD3‐enhanced AKT (S473) phosphorylation (Figure 4A). The level of either mTOR or RAPTOR remained unchanged; however, RICTOR changed along with NCAPD3 in DU145 and LNCaP cells (Figure 4B). In brief, changing the level of NCAPD3 affected the composition of the mTORC2 complex, which in turn changed in its kinase activity. Moreover, changes in the phosphorylation of mTORC2 downstream protein PKC also demonstrated this point (Figure S2A). Following the further activation of AKT, its downstream target mTORC1 was activated, resulting in the phosphorylation of 4E‐BP1 and P70S6K (Figure S2A). Similar results were also obtained in the non‐transformed human prostate epithelial cell line (BPH‐1) with NCAPD3 overexpression (Figure S2B).

Previous studies have reported that NSD2 (termed WHSC1 or MMET) regulated RICTOR expression and was a downstream factor of EZH2 in PCa.^19^, ^20^ GSK126 was a highly selective EZH2 inhibitor, and the descending expression of H3K27me3 showed that the inhibitory effect on EZH2 was enhanced following the increase in the concentration of GSK126 (Figure S2C). The same trend was observed in NSD2, RICTOR, and p‐AKT (S473). Interestingly, our published study confirmed that EZH2 and STAT3 were induced by NCAPD3, and STAT3 was the potential transcription factor to regulate EZH2.^21^ NCAPD3 indeed promoted STAT3, EZH2, and NSD2 expression, but this promotion was potently inhibited by GSK126, and RICTOR and p‐AKT (S473) also changed as them (Figure 4C). Similarity, after STAT3 knockdown in DU145 cells, NCAPD3‐enhanced expression of EZH2, NSD2, and RICTOR were obviously attenuated (Figure 4D, Figure S2D,E). Immunofluorescence staining assay and nucleocytoplasmic separation assay showed that STAT3 and NCAPD3 mainly co‐localized in the nucleus (Figure 4E and Figure S2F), and co‐IP data showed that NCAPD3 interacted with STAT3 in both DU145 and LNCaP cells (Figure 4F; NCAPH2 as the positive control). Furthermore, ChIP‐assay data suggested that more STAT3 was recruited to the EZH2 promoter in DU145 cells with NCAPD3 overexpression, while the result was reversed in NCAPD3‐knockdown LNCaP cells (Figure 4G). Together, these data implicated that NCAPD3‐enhanced phosphorylation of AKT (S473) in PCa cells was mediated by STAT3/EZH2/NSD2/mTORC2 axis.

### 3.5 NCAPD3 enhances the positive mutual activation between STAT3 and JAK2 in PCa cells

**FIGURE 5:**
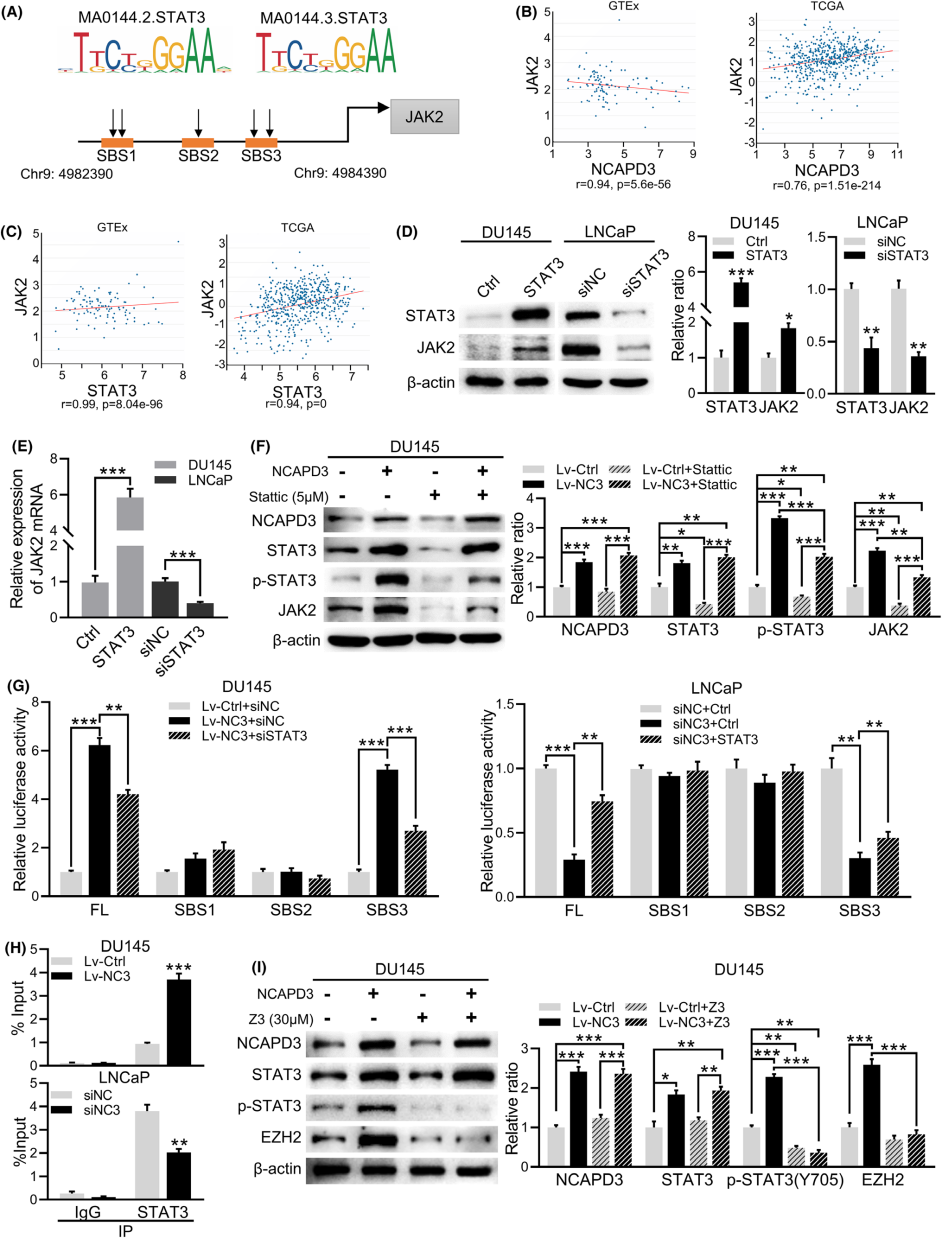
NCAPD3 enhanced the mutual activation between STAT3 and JAK2. (A) Prediction results of STAT3 binding sites and binding sequences on JAK2 promoter. Three binding sites were termed as SBS1, SBS2, and SBS3, respectively. (B and C) NCAPD3, STAT3, and JAK2 expression levels in TCGA tumor tissues and GTEx normal tissues. (D and E) Expression levels of JAK2 (including protein and mRNA) were measured by western blot and qRT‐PCR in PCa cells with transfection of STAT3 or siSTAT3. (F) Protein levels of NCAPD3, STAT3, p‐STAT3 (Y705), and JAK2 in DU145 cells with transfection and treatment as indicated in figures. (G) Results of dual‐luciferase assays for evaluating the NCAPD3‐enhanced binding and activation of STAT3 on the promoter of JAK2. FL, full long of JAK2 promoter. SBS1, SBS2, and SBS3 represented three predicted STAT3 binding sites on the JAK2 promoter, respectively. (H) Enrichment of STAT3 on the JAK2 promoter in PCa cells transfected as indicated in figures. IgG served as a nonspecific control. (I) Western blot analysis of proteins in DU145 cells overexpressed with NCAPD3 and treated with Z3 (5 μM) as indicated in the figure. Results showed as mean ± SD, *p < 0.05; **p < 0.01; ***p < 0.001; ns, not significant.

To explore the mechanism by which NCAPD3 upregulated JAK2 expression, we predicted the transcription factors binding to the JAK2 promoter using the JASPAR. Three possible binding sites (SBS1, SBS2, and SBS3) indicated that STAT3 was most likely the potential transcription factor of JAK2 (Figure 5A). Then, we compared the expression between JAK2 and NCAPD3 or STAT3 in GTEx normal tissues and TCGA tumor tissues, and found both NCAPD3 and STAT3 showed strong positive correlations with JAK2 in tumor tissues (Figure 5B,C). WB and qRT‐PCR showed that overexpression of STAT3 increased JAK2, while knockdown of STAT3 decreased JAK2 (Figure 5D,E). A STAT3 inhibitor (Stattic) blocked NCAPD3‐induced upregulation of JAK2 (Figure 5F). To identify the binding site of STAT3 in JAK2 promoter, four luciferase reporter plasmids were constructed, including full‐length of JAK2 promoter (JAK2‐Luc‐FL), the fragment of SBS1 (JAK2‐Luc‐SBS1), SBS2 (JAK2‐Luc‐SBS2), and SBS3 (JAK2‐Luc‐SBS3), respectively. Relative luciferase activity (RLA) of DU145 cells transfected with JAK2‐Luc‐FL was increased compared to the control and further increased when co‐transfected with STAT3 (Figure S3A). Co‐transfected with siSTAT3 impaired the RLA rise by JAK2‐FL in LNCaP. Only the RLA of the JAK2‐Luc‐SBS3 group enhanced when transfecting with NCAPD3 and dropped when co‐transfecting with NCAPD3 and siSTAT3 (Figure 5G). So, we considered that fragment SBS3 was the key binding site of STAT3 in the JAK2 promoter. ChIP‐PCR suggested that overexpression of NCAPD3 could promote the binding of STAT3 at the JAK2 promoter, whereas NCAPD3 knockdown in LNCaP cells impaired this binding (Figure 5H).

As we know, JAK2/STAT3 axis plays an essential role in promoting tumor initiation and development, and JAK2 is the directly upstream kinase of STAT3.^22^ NCAPD3 enhanced STAT3 phosphorylation at residue Y705, and this enhancement could be restrained by JAK2 inhibitor (Z3) (Figure 5F,I). Thus, there was a positive mutual activation between STAT3 and JAK2 in PCa cells, and NCAPD3 could enhance this mutual activation by increasing the expression of STAT3. It should be noted that NCAPD3 overexpression can not enhance the phosphorylation of AKT and the expression of JAK2 and EZH2 in PC‐3 cells, which do not express STAT3 (Figure S3B,C). However, all of them were remarkably increased when wild‐type STAT3 overexpressed in PC‐3. This suggested that NCAPD3‐induced STAT3 high expression was the central step in its promotion of the phosphorylation of AKT and the expression of JAK2 and EZH2.

### 3.6 NCAPD3 promotes PCa xenograft tumor growth via phosphorylating AKT in vivo

**FIGURE 6:**
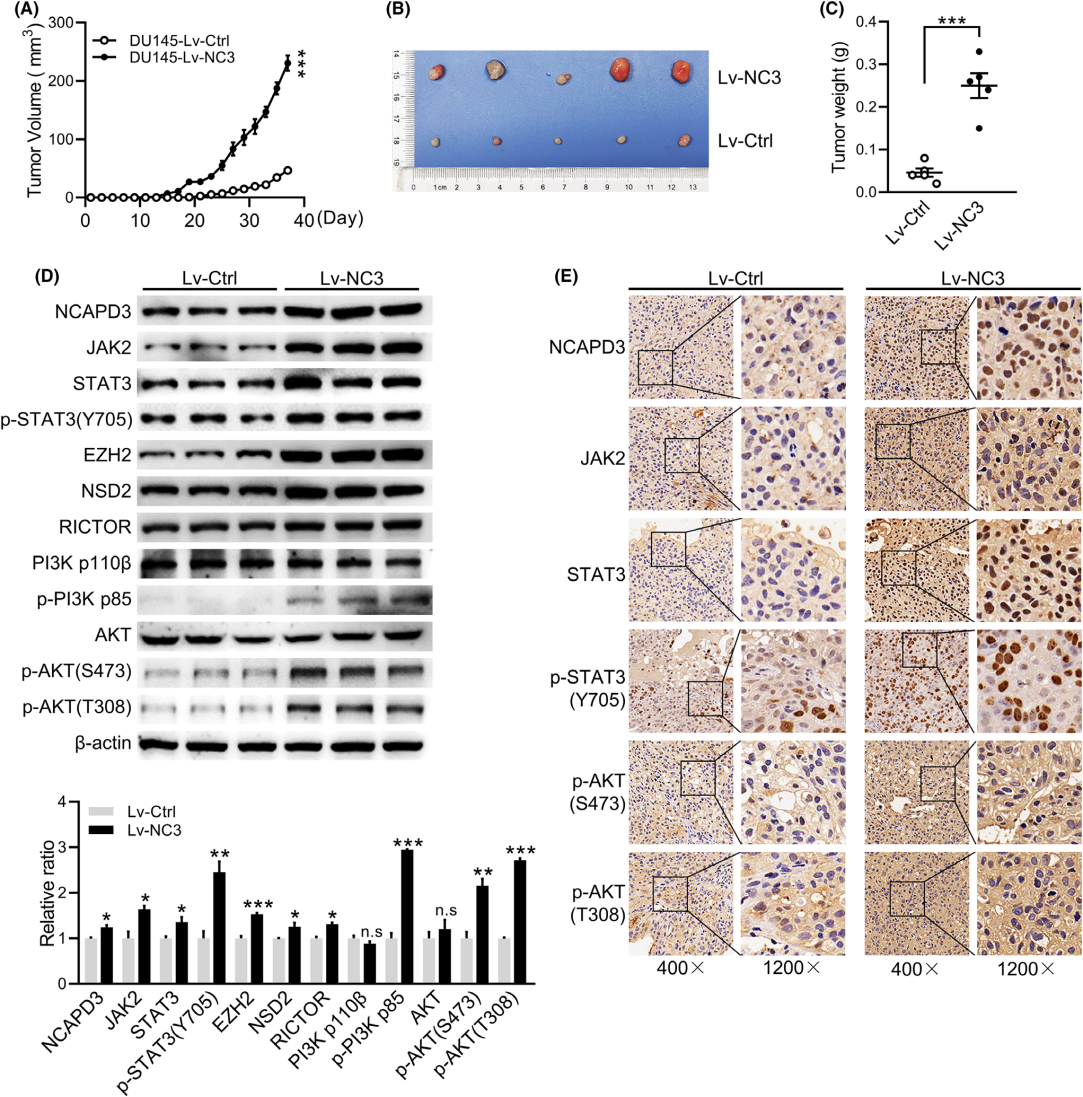
NCAPD3 promoted PCa xenograft tumor growth via activation of AKT in vivo. (A) Tumor growth curves were analyzed with the daily tumor volume measured data. (B) Photographs of dissected tumors from nude mice subcutaneously injected with stable NCAPD3‐overexpression DU145 cells and control cells. (C) Average tumor weight of each group. (D) Western blot analysis of proteins in the xenograft tissues from NCAPD3‐overexpression and control group mice. (E) Immunohistochemistry assays of proteins in xenograft tissues. Representative images showed with 400× and 1200×. Statistically significance indicated: *p < 0.05; **p < 0.01; ***p < 0.001; Student's *t*‐test.

To verify the results in vitro, DU145 cells stably overexpressing NCAPD3 were constructed and subcutaneously injected into nude mice. Compared with the control group, the volume and weight of tumors were higher in the NCAPD3‐overexpressing group, and the body weight had no significant difference (Figure 6A–C and Figure S3D). The protein levels of JAK2, STAT3, EZH2, RICTOR, and the phosphorylation levels of STAT3 (Y705), PI3K, AKT (S473), and AKT (T308) were upregulated in NCAPD3 overexpression xenograft tumor, while the total protein levels of PI3K and AKT had no changes (Figure 6D). Furthermore, IHC analysis showed that NCAPD3, STAT3, and p‐STAT3 (Y705) in the nucleus and JAK2, p‐AKT (S473), and p‐AKT (T308) in the cytoplasm was higher in the NCAPD3 overexpression group than in the control group (Figure 6E). These results suggested that NCAPD3 promoted subcutaneous xenograft tumor growth which might be attributed to activating AKT.

## 4 DISCUSSION

**FIGURE 7:**
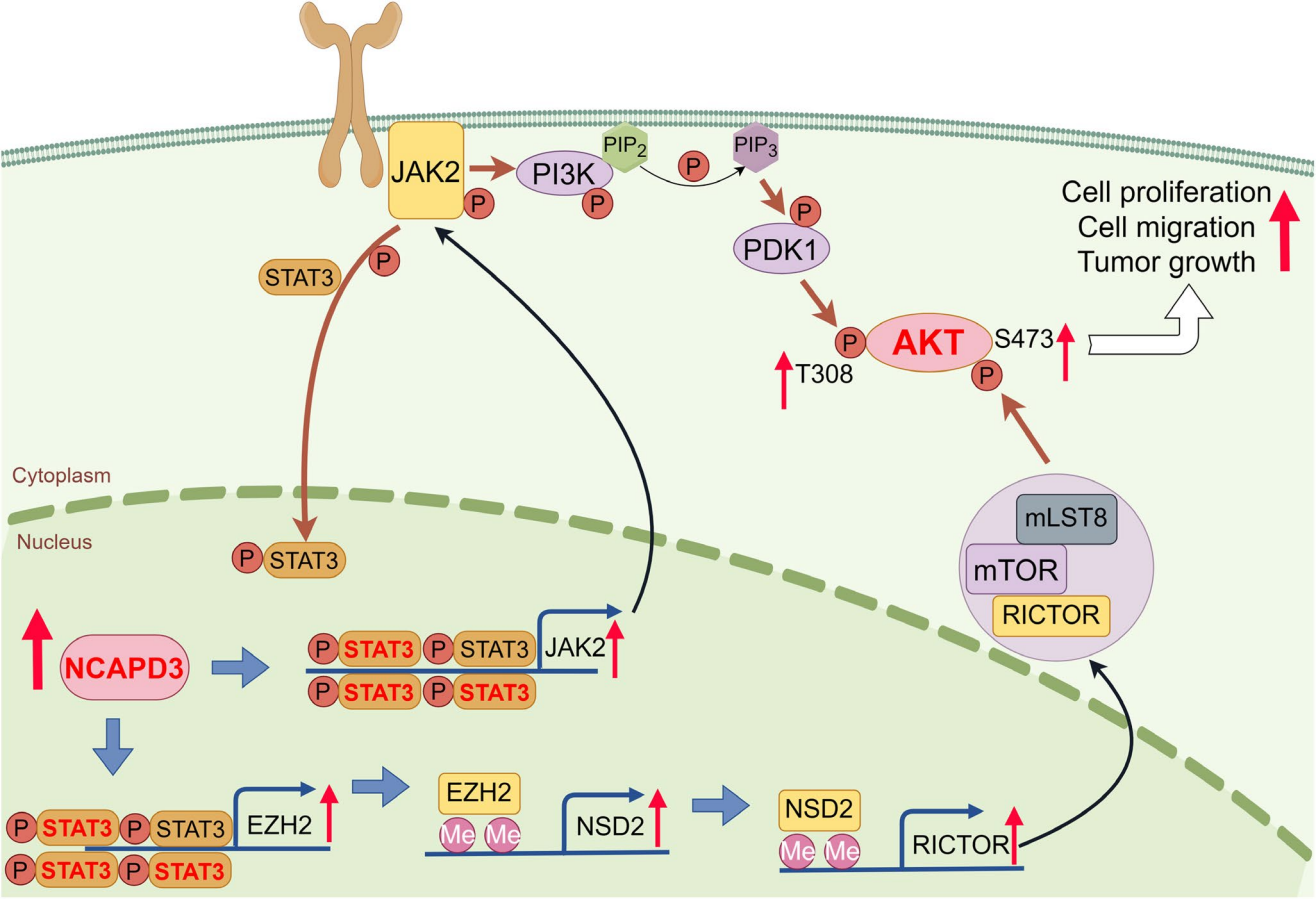
NCAPD3 increased the level of STAT3 and recruited more STAT3 to the promoters of EZH2 and JAK2 and then activated EZH2/NSD2/mTORC2 and JAK2/PI3K pathways, which phosphorylated AKT at Thr 308 and Ser 473. Moreover, there is a positive mutual activation between STAT3 and JAK2, further enhanced by NCAPD3 to promote PCa progression. The graph was drawn by Figdraw.

Although non‐SMC family members of condensins are considered to play an important role in many cellular physiological processes of human disease, especially in regulating mitosis and gene expression, little is known about their molecular mechanism.^7^, ^23^ Previous studies report that NCAPD2 plays an essential role in colorectal tumorigenesis and the progression of colorectal cancer.^24^ In hepatocellular carcinoma, the knockdown of NCAPG2 arrests tumor cells in the G1/S phase and reduces their migration and invasion abilities.^25^ In addition, NCAPD3‐induced activation of IKK/NF‐κB pathway and secretion of proinflammatory cytokines TNF‐α/IL‐6 promote the development of ulcerative colitis.^26^ We have reported that NCAPD3 has higher expression in prostate cancer and is involved in androgen receptor‐promoted PCa progression.^27^ In this study, we verified that NCAPD3 could activate JAK2/PI3K and EZH2/NSD2/mTORC2 axes by upregulating the expression of STAT3, which finally resulted in increasing the phosphorylation levels of AKT (T308 and S473) in PCa cells. Furthermore, we found that STAT3 and JAK2 were mutually activated in PCa cells, and NCAPD3 could enhance this positive mutual activation by upregulating STAT3 expression and phosphorylation (Figure 7).

AKT is frequently dysregulated in many types of cancers, such as colon cancer, ovarian cancer, and breast cancer.^28^ AKT is predominantly phosphorylated by kinases PDK1 and mTORC2 at Thr308 and Ser473, which induces the activation of AKT.^17^ As a transcriptional factor, STAT3 has been reported to upregulate EZH2 expression and associate with advanced TNM stage and poor prognosis in gastric cancer.^29^ EZH2 and NSD2 are members of histone methyltransferase families and are separately responsible for catalyzing H3K27me3 and H3K36me2. Recent reports suggest that EZH2 and NSD2 are overexpressed and exert oncogenic effects in multiple cancers.^20^ In PCa, coordinated expression of EZH2 and NSD2 is found to promote tumor progression.^19^ NSD2 significantly upregulates the expression of RICTOR by reducing H3K36me2 levels.^21^ In this study, we reported that NCAPD3 increased the expression of STAT3, and activated EZH2 and JAK2 transcription by promoting STAT3 to bind the promoter of them. Overexpression of EZH2 and JAK2 led to abnormal activation of EZH2/NSD2/mTORC2 and JAK2/PI3K pathway, eventually presenting a large increase in the hyperphosphorylation of AKT.

JAK2 directly phosphorylates and activates STAT3, which are both involved in diverse cellular processes, such as cell proliferation, migration, and autophagy.^30^ However, no studies reported the transcriptional effect of STAT3 on the expression of JAK2. In this study, we verified for the first time that NCAPD3 recruited more STAT3 to the promoter of JAK2 gene to activate the transcription of JAK2 in PCa. Therefore, we confirmed for the first time that there was a mutual activation between STAT3 and JAK2, which was further enhanced by NCAPD3 in NCAPD3‐promoted progression of PCa.

In conclusion, we gain a deeper understanding of the molecular mechanisms of NCAPD3 promoting PCa development by STAT3 and AKT. NCAPD3 increases the expression of STAT3 and recruits more STAT3 to the promoters of JAK2 and EZH2 genes in PCa, and then results in the upregulation of AKT phosphorylation (T308/S473) via activating JAK2/PI3K and EZH2/NSD2/mTORC2 pathways. To our pleasant surprise, we found and preliminarily proved that the reciprocal regulation between STAT3 and JAK2 in DU145 and LNCaP, and NCAPD3 appeared to be playing a significant function here. Taking this report together with our published literature, we proposed that NCAPD3 affected different stages of PCa through specific pathways, and these are still required to be confirmed by more high‐quality experiments, which could help to broaden the therapeutic strategies for prostate cancer.

## AUTHOR CONTRIBUTIONS

YZ, PL, QZ, and SL designed the project and experiments. YZ, WX, FY, JR, and YW performed the experiments. YZ, SL, and PL integrated and analyzed the data. YZ, WX, ZX, and PL wrote and edited the manuscript. All authors read and approved the final version of the manuscript.

## FUNDING INFORMATION

National Natural Science Foundation of China (NNSFC) (grant no. 81872104 and 81472415). Taizhou Science and Technology Support Plan (Social Development) Project (SSF20230019).

## DISCLOSURES

Authors declare that there are no any conflicts of interest that could influence the research reported in the present study.

## Supporting information


Figures S1–S3.

Tables S1–S3.

## Data Availability

The supporting data of the current study are available from the corresponding author upon reasonable request.
