# Supplementary material for: NCAPD3‐mediated AKT activation regulates prostate cancer progression
Source: FASEB Bioadv. 2025 Jan 7;7(2):e1488. doi: 10.1096/fba.2024-00073 (PMC11795278; doi:10.1096/fba.2024-00073)
Supplement: Supplementary file 1 — Figures S1–S3. [file FBA2-7-e1488-s002.docx]

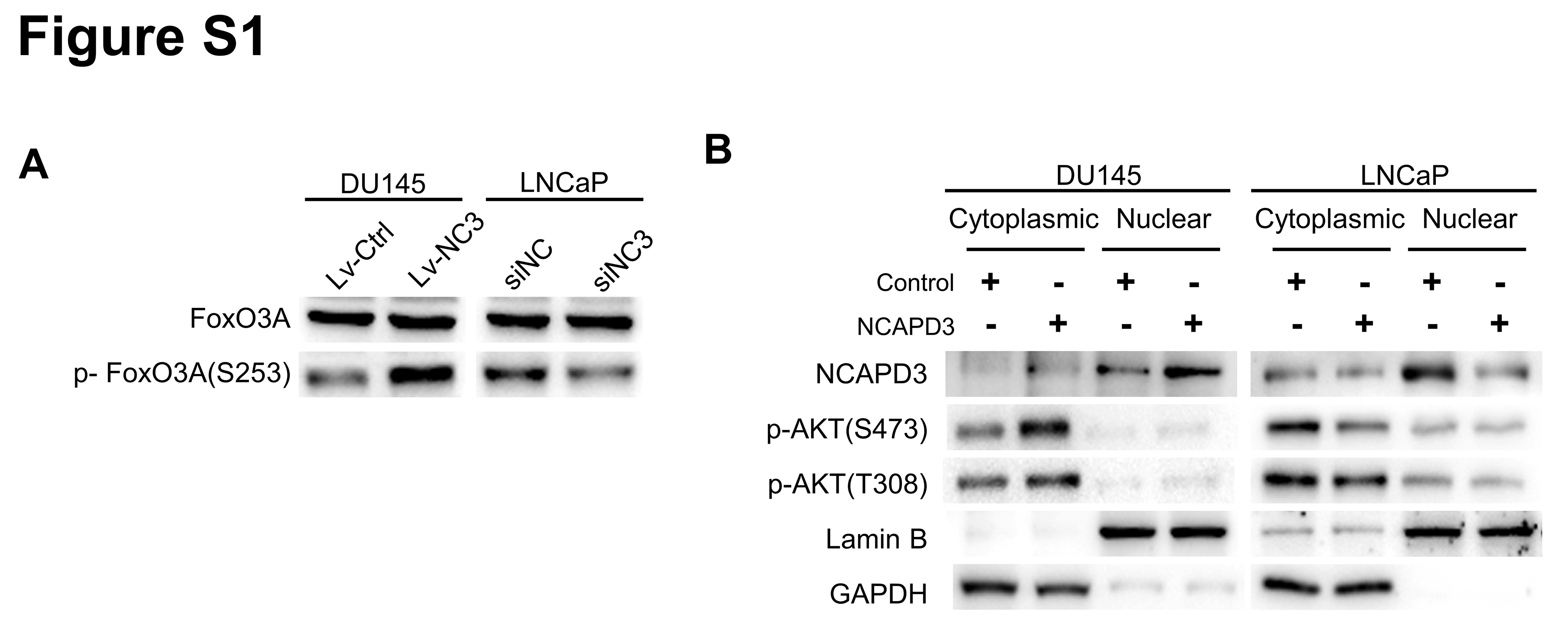


**Figure S1.** (A) Phosphorylation level of FoxO3A (S253) and its total protein level in NCAPD3 overexpression or NCAPD3 knockdown PCa cells. (B) Levels of NCAPD3, p-AKT (S473) and p-AKT (T308) in nucleus and cytoplasm detected by nucleocytoplasmic separation assay. Lamin B and GAPDH served as the internal controls in the nucleus and cytoplasm, respectively.


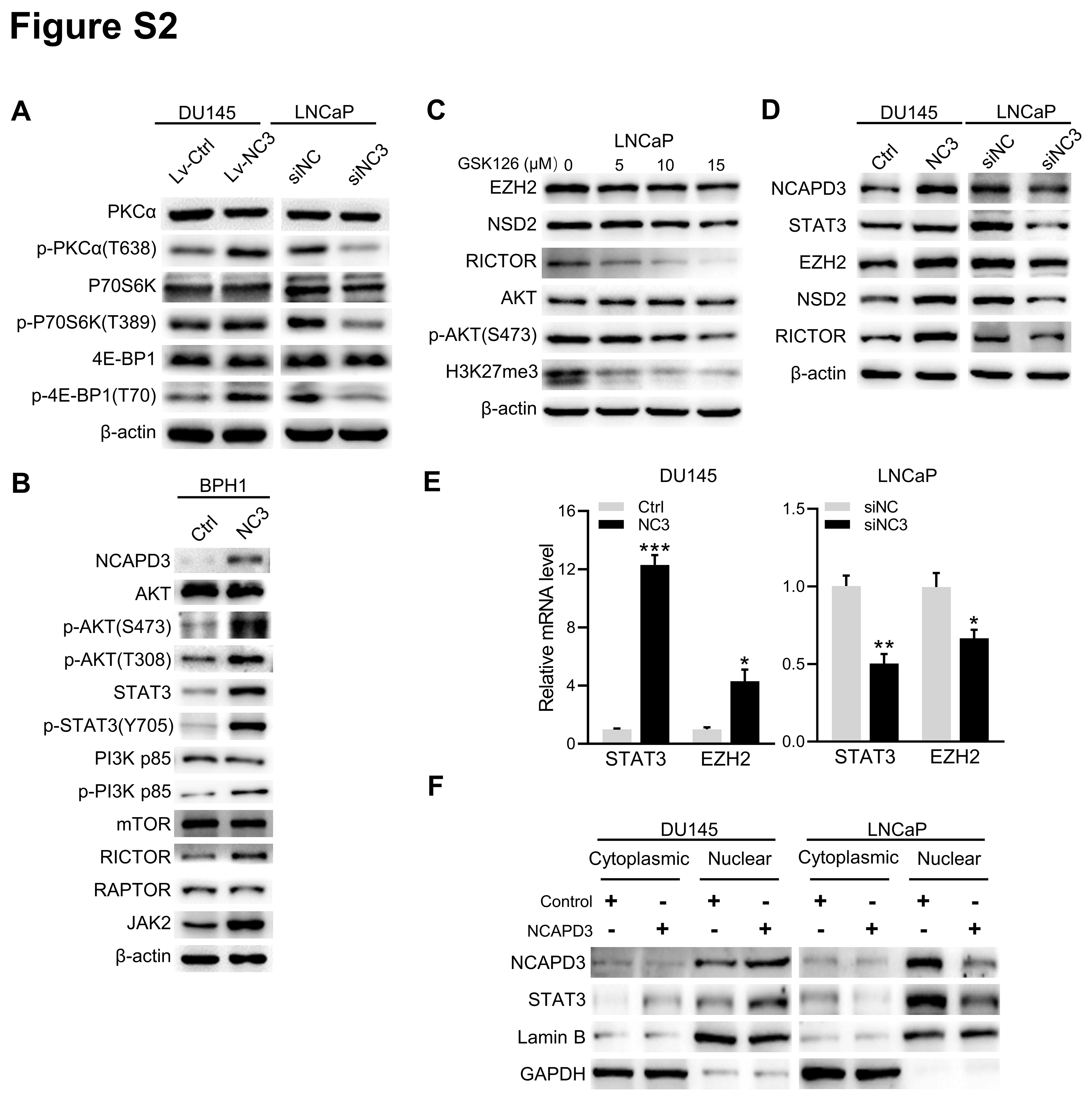


**Figure S2.** (A) Protein expression of PKCα, p-PKCα(T638), P70S6K, p-P70S6K(T389), 4E-BP1 and p-4E-BP1(T70) in NCAPD3 overexpression or NCAPD3 knockdown PCa cells. (B) The protein level of genes in the non-transformed human prostate epithelial cell line (BPH-1) was determined by WB after overexpressing NCAPD3.(C) Expression of EZH2, NSD2, RICTOR, AKT and p-AKT (S473) in LNCaP cells treated with GSK126 (0, 5, 10, 15 μM) (H3K27me3 as the control). (D, E) Expression of STAT3, EZH2, NSD2 and RICTOR assayed by western blot and RT-qPCR in PCa cells with NCAPD3 overexpression or knockdown. (F) Levels of NCAPD3 and STAT3 in cytoplasm and nucleus of PCa cells with NCAPD3 overexpression and knockdown (assayed by western blot, GAPDH and Lamin B as the controls). Values are means±S.E. from n=3 independent repetitions, *P < 0.05, **P < 0.01, ***P < 0.001, based on Student's t-test.


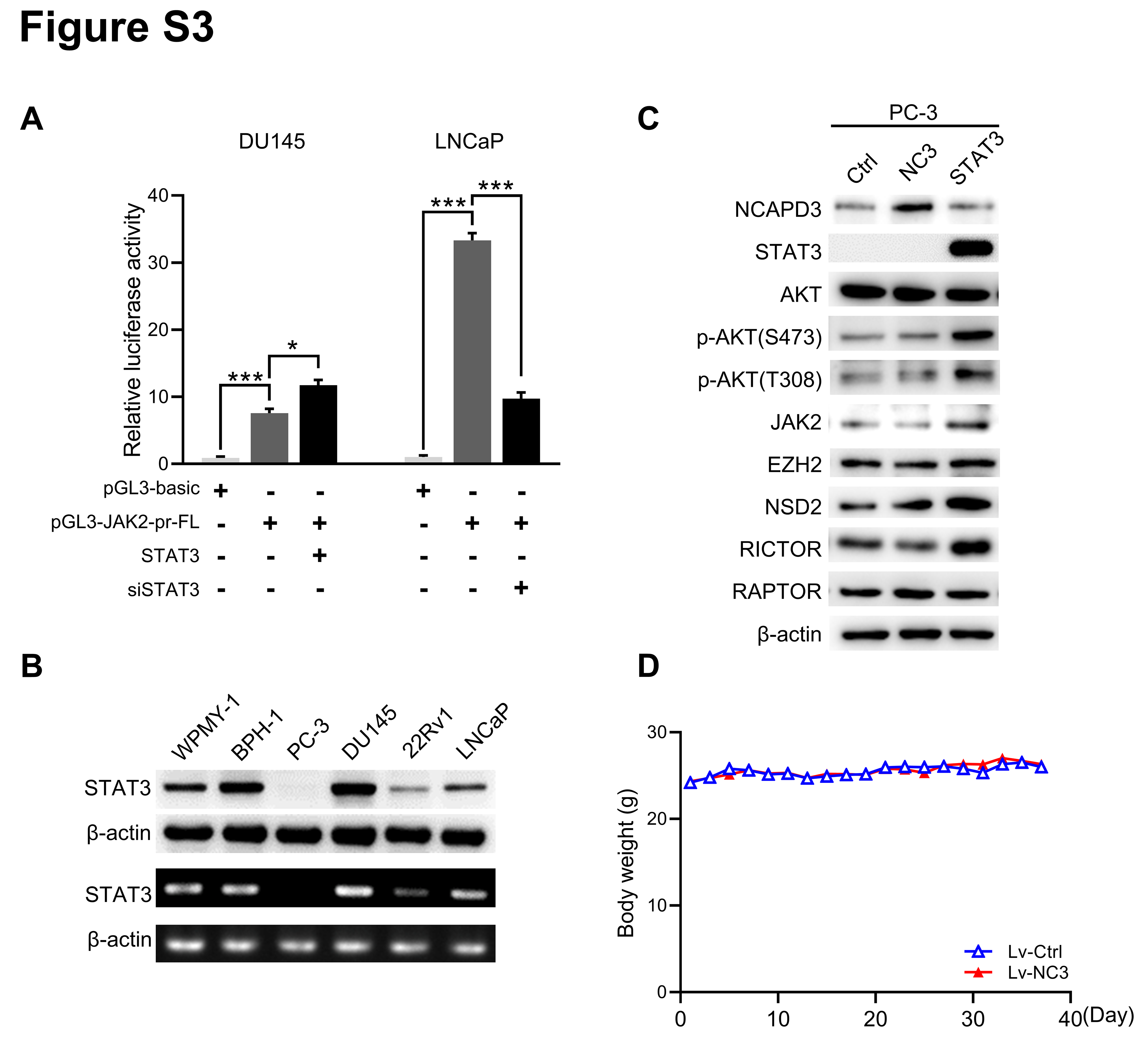


**Figure S3.** (A) Dual-luciferase assays to evaluate STAT3 binding to the promoter and enhancing the expression of the JAK2 gene in DU145 and LNCaP cells. The pGL3-basic served as a control plasmid. *P < 0.05, **P < 0.01, ***P < 0.001, based on Student's t-test. (B) Protein and mRNA levels of STAT3 in WPMY-1, BPH-1, PC-3, DU145, 22Rv1 and LNCaP examined by western blotting and PCR. (C) Western blot analysis of proteins in PC-3 cells with transfection of NCAPD3 or STAT3. (D) Changes in body weight of the nude mice during the growth of xenograft. Statistics of mouse body weight. There was no significant difference in the average body weight of the two groups.
