# Supplementary material for: NCAPD3‐mediated AKT activation regulates prostate cancer progression
Source: FASEB Bioadv. 2025 Jan 7;7(2):e1488. doi: 10.1096/fba.2024-00073 (PMC11795278; doi:10.1096/fba.2024-00073)
Supplement: Supplementary file 2 — Tables S1–S3. [file FBA2-7-e1488-s001.docx]

Supplementary tables

Table S1. The sequence of siRNA oligonucleotides targeting NCAPD3, STAT3 and negative control.

| Gene symbol | siRNA sequence（5’to3’） |
| --- | --- |
| NCAPD3 | AGGAAUUCAAGUUAACAGAGGCUUG |
| STAT3 | GCAACAGAUUGCCUGCAUUTT |
| Negative control | UUCUCCGAACGUGUCACGUTT |

Abbreviations: siRNA, small interfering RNA. NCAPD3, non-SMC condensin II complex subunit D3. STAT3, signal transducer and activator of transcription 3.

Table S2. Primers for RT-qPCR

| Gene symbol | Primer sequence（5’to3’） |
| --- | --- |
| β-actin-F | CACCATTGGCAATGAGCGGTTC |
| β-actin-R | AGGTCTTTGCGGATGTCCACGT |
| NCAPD3-F | GAAAGCCAGGAACTGAGCCGAT |
| NCAPD3-R | AGCCAGCAATCTTGGAGAGCAG |
| STAT3-F | CAGCAGCTTGACACACGGTA |
| STAT3-R | AAACACCAAAGTGGCATGTGA |
| EZH2-F | AAAGGAGTTTGCTGCTGCTC |
| EZH2-R | TGTTATTGGGAAGCCGTCCT |
| JAK2-F | TGCCGGTATGACCCTCTACA |
| JAK2-R | ACCAGCACTGTAGCACACTC |

Abbreviations: RT-qPCR, reverse transcriptase quantitative polymerase chain reaction. NCAPD3, non-SMC condensin II complex subunit D3. STAT3, signal transducer and activator of transcription 3. EZH2, enhancer of zeste homolog 2. JAK2, Janus kinase 2. F, forward. R, reverse.

Table S3. Primers for CHIP-qPCR

| Gene symbol | Primer sequence（5’to3’） |
| --- | --- |
| EZH2 promoter for STAT3 (F) | CGGGGTACCAACTGGTTCAAACTTGGCTTCCAGC |
| EZH2 promoter for STAT3 (R) | CCGCTCGAGCAGCCCAATCGCCATCGCTTTTAT |
| JAK2 promoter for STAT3 (F) | TCTCAAGGTCTTTGCCTTCAG |
| JAK2 promoter for STAT3 (R) | GGGTGTGCTATTTTACGGTGG |

Abbreviations: CHIP-qPCR, Chromatin Immunoprecipitation quantitative PCR. STAT3, signal transducer and activator of transcription 3. EZH2, enhancer of zeste homolog 2. JAK2, Janus kinase 2. F, forward. R, reverse.
